# [Diphenyldi(pyrazol-1-yl)methane]­dinitratocobalt(II)

**DOI:** 10.1107/S1600536810000565

**Published:** 2010-01-13

**Authors:** Janet L. Shaw, Bruce C. Noll

**Affiliations:** a Kennesaw State University, 1000 Chastain Road, Kennesaw, GA 30144-5591, USA; b Bruker AXS Inc., 5465 East Cheryl Parkway, Madison, WI 53711, USA

## Abstract

In the title compound, [Co(NO_3_)_2_(C_19_H_16_N_4_)], the diphenyl­dipyrazolylmethane ligand coordinates to Co^II^ in a bidentate fashion forming a six-membered ring with an approximate boat configuration. The mean planes of the two pyrazolyl rings are separated by an angle of 39.6 (2)°. The coordination at the Co^II^ center is best described as distorted octa­hedral with two NO_3_^−^ anions serving as bidentate ligands for charge balance. The dihedral angle between the mean planes of the two nitrate rings is 85.0 (1)° and that between the mean planes of the two phenyl rings is 73.7 (1)°. The crystal structure is stabilized by weak inter­molecular C—H⋯O and intra­molecular C—H⋯N hydrogen-bond inter­actions.

## Related literature

For related structures incorporating diphenyl­dipyrazolyl­methane ligands, see: Shiu *et al.* (1993 ▶); Tsuji *et al.* (1999 ▶); Reger *et al.* (2004 ▶); Shaw *et al.* (2004 ▶, 2005 ▶, 2009 ▶); Baho & Zargarian (2007*a* ▶,*b* ▶).
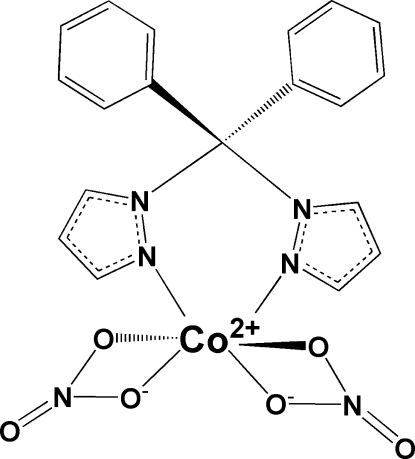


## Experimental

### 

#### Crystal data

[Co(NO_3_)_2_(C_19_H_16_N_4_)]
*M* *_r_* = 483.31
Monoclinic, 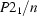

*a* = 8.5476 (14) Å
*b* = 14.8058 (17) Å
*c* = 16.818 (3) Å
β = 103.383 (4)°
*V* = 2070.6 (5) Å^3^
*Z* = 4
Mo *K*α radiation
μ = 0.88 mm^−1^
*T* = 200 K
0.50 × 0.30 × 0.30 mm

#### Data collection

Bruker SMART X2S benchtop diffractometer
Absorption correction: multi-scan (*SADABS*; Sheldrick, 2008*a* ▶) *T*_min_ = 0.668, *T*_max_ = 0.778
13223 measured reflections
3666 independent reflections
3042 reflections with *I* > 2σ(*I*)
*R*_int_ = 0.035

#### Refinement

*R*[*F* ^2^ > 2σ(*F* ^2^)] = 0.035
*wR*(*F* ^2^) = 0.118
*S* = 0.96
3666 reflections
289 parameters
H-atom parameters constrained
Δρ_max_ = 0.42 e Å^−3^
Δρ_min_ = −0.45 e Å^−3^

### 

Data collection: *APEX2* (Bruker, 2009 ▶); cell refinement: *APEX2* and *SAINT* (Bruker, 2009 ▶); data reduction: *SAINT* and *XPREP* (Bruker, 2008 ▶); program(s) used to solve structure: *SHELXS97* (Sheldrick, 2008*b* ▶); program(s) used to refine structure: *SHELXL97* (Sheldrick, 2008*b* ▶); molecular graphics: *SHELXTL* (Sheldrick, 2008*b* ▶); software used to prepare material for publication: *publCIF* (Westrip, 2010 ▶).

## Supplementary Material

Crystal structure: contains datablocks global, I. DOI: 10.1107/S1600536810000565/jj2017sup1.cif

Structure factors: contains datablocks I. DOI: 10.1107/S1600536810000565/jj2017Isup2.hkl

Additional supplementary materials:  crystallographic information; 3D view; checkCIF report

## Figures and Tables

**Table 1:** Hydrogen-bond geometry (Å, °)

*D*—H⋯*A*	*D*—H	H⋯*A*	*D*⋯*A*	*D*—H⋯*A*
C17—H17⋯O5^i^	0.93	2.54	3.413 (3)	157
C10—H10⋯O3^ii^	0.93	2.59	3.399 (4)	146
C3—H3⋯O4^iii^	0.93	2.50	3.313 (3)	146
C19—H19⋯N1	0.93	2.46	2.799 (3)	102

Symmetry codes: (i) 
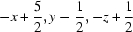
; (ii) 
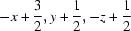
; (iii) 
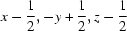
.

**Fig. 1.**
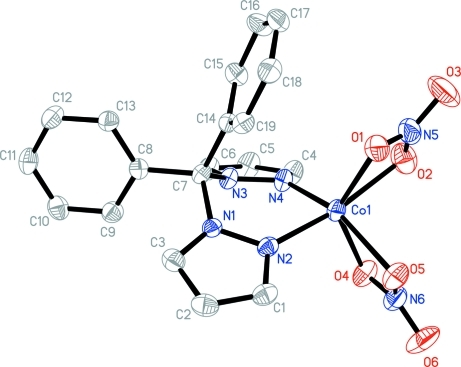
The molecular structure of Co(C19H16N4)(NO3)2 with 50% thermal ellipsoids. Hydrogen atoms have been omitted for clarity.

**Fig. 2.**
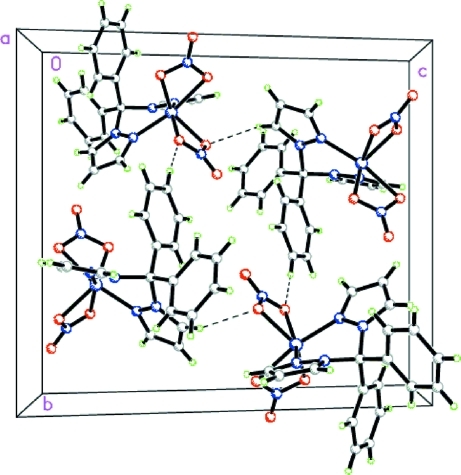
The packing diagram for Co(C19H16N4)(NO3)2 viewed along the a axis. Dashed lines indicate weak C—H···O intermolecular hydrogen bond interactions.

## supplementary crystallographic information

### Comment

The metal chemistry of diphenyldipyrazolylmethane ligands was first explored by Shiu *et al.* (1993) who crystallized two complexes of the 3,5-dimethylpyrazolyl variant with molybdenum. Similar complexes with Pd^II^ were synthesized by Tsuji *et al.* (1999) and Reger *et al.* (2004) who generated complexes with Ag^I^ . More recently, compounds with diphenyldipyrazolylmethane ligands complexed with Cu^I/II^ (Shaw *et al.* 2004;2005), Ni^II^ (Baho & Zargarian, 2007*a*; 2007*b*), and Zn^II^ (Shaw *et al.* 2009) have appeared in the literature.

In the title compound, Co(C_19_H_16_N_4_)(NO_3_)_2_, the diphenyldipyrazolylmethane ligand coordinates to the Co^II^ in a bidentate fashion forming a six-membered ring with an approximate boat configuration (Fig. 1). The mean planes of the two pyrazolyl rings are separated by 39.55 (12)°. The geometry at the Co^II^ is best described as a distorted octahedral with two NO_3_^-^ anions serving as bidentate ligands for charge balance.  The N2 and N4 atoms are the bidentate groups that form a heteroscorpionate type structure coordinated to a d^2^sp^3^ hybridized Co^II ^ion.  The dihedral angle between the mean planes of the two nitrato rings is 84.52 (10)° and between the mean planes of the two phenyl rings is 73.71 (6)°.  The crystal structure is stabilized by weak intermolecular C—H···O and intramolecular C—H···N hydrogen bond interactions (Fig. 2; Table 1).

### Experimental

The title compound was prepared by reacting cobalt(II) nitrate hexahydrate (1.64 mmoles) with diphenyldipyrazolylmethane (1.97 mmoles) in ethanol (100 ml). After 24 h of stirring, the solution was evaporated under reduced pressure to afford a red solid. Crystals were isolated by redissolving the solid in dichloromethane and layering with hexanes.

### Refinement

All hydrogen atoms were refined using a riding model. C—H values were set from 0.93 to 0.97 Å with *U*_iso_(H) = 1.2*U*_eq_(C).

### Crystal data

**Table:**

[Co(NO_3_)_2_(C_19_H_16_N_4_)]	*F*(000) = 988
*M**_r_* = 483.31	*D*_x_ = 1.550 Mg m^−^^3^
Monoclinic, *P*2_1_/*n*	Mo *K*α radiation, λ = 0.71073 Å
Hall symbol: -P 2yn	Cell parameters from 5780 reflections
*a* = 8.5476 (14) Å	θ = 2.5–24.7°
*b* = 14.8058 (17) Å	µ = 0.88 mm^−^^1^
*c* = 16.818 (3) Å	*T* = 200 K
β = 103.383 (4)°	Block, red
*V* = 2070.6 (5) Å^3^	0.50 × 0.30 × 0.30 mm
*Z* = 4	

### Data collection

**Table:**

Bruker SMART X2S benchtop diffractometer	3666 independent reflections
Radiation source: microfocus sealed tube	3042 reflections with *I* > 2σ(*I*)
doubly curved silicon crystal	*R*_int_ = 0.035
ω scans	θ_max_ = 25.1°, θ_min_ = 1.9°
Absorption correction: multi-scan (*SADABS*; Sheldrick, 2008a)	*h* = −10→10
*T*_min_ = 0.668, *T*_max_ = 0.778	*k* = −13→17
13223 measured reflections	*l* = −19→19

### Refinement

**Table:**

Refinement on *F*^2^	Primary atom site location: structure-invariant direct methods
Least-squares matrix: full	Secondary atom site location: difference Fourier map
*R*[*F*^2^ > 2σ(*F*^2^)] = 0.035	Hydrogen site location: inferred from neighbouring sites
*wR*(*F*^2^) = 0.118	H-atom parameters constrained
*S* = 0.96	*w* = 1/[σ^2^(*F*_o_^2^) + (0.0648*P*)^2^ + 0.2696*P*] where *P* = (*F*_o_^2^ + 2*F*_c_^2^)/3
3666 reflections	(Δ/σ)_max_ = 0.001
289 parameters	Δρ_max_ = 0.42 e Å^−^^3^
0 restraints	Δρ_min_ = −0.45 e Å^−^^3^

### Special details

**Table:**

Geometry. All esds (except the esd in the dihedral angle between two l.s. planes) are estimated using the full covariance matrix. The cell esds are taken into account individually in the estimation of esds in distances, angles and torsion angles; correlations between esds in cell parameters are only used when they are defined by crystal symmetry. An approximate (isotropic) treatment of cell esds is used for estimating esds involving l.s. planes.
Refinement. Refinement of *F*^2^ against ALL reflections. The weighted *R*-factor *wR* and goodness of fit *S* are based on *F*^2^, conventional *R*-factors *R* are based on *F*, with *F* set to zero for negative *F*^2^. The threshold expression of *F*^2^ > σ(*F*^2^) is used only for calculating *R*-factors(gt) *etc*. and is not relevant to the choice of reflections for refinement. *R*-factors based on *F*^2^ are statistically about twice as large as those based on *F*, and *R*- factors based on ALL data will be even larger.

### Fractional atomic coordinates and isotropic or equivalent isotropic displacement parameters (Å^2^)

**Table:**

	*x*	*y*	*z*	*U*_iso_*/*U*_eq_	
Co1	1.03355 (4)	0.17196 (2)	0.34055 (2)	0.02457 (16)	
N1	0.7926 (2)	0.22829 (13)	0.18460 (12)	0.0213 (5)	
N2	0.9396 (3)	0.24461 (14)	0.23606 (13)	0.0235 (5)	
N3	0.6768 (2)	0.14506 (15)	0.28100 (13)	0.0237 (5)	
N4	0.8088 (2)	0.13421 (15)	0.34433 (13)	0.0265 (5)	
C1	1.0006 (3)	0.31437 (17)	0.20321 (17)	0.0291 (6)	
H1	1.0991	0.3413	0.2258	0.035*	
C2	0.8968 (4)	0.34157 (18)	0.13031 (18)	0.0318 (6)	
H2	0.9123	0.3885	0.0961	0.038*	
C3	0.7677 (3)	0.28493 (17)	0.11973 (16)	0.0278 (6)	
H3	0.6786	0.2852	0.0758	0.033*	
C4	0.7511 (3)	0.1164 (2)	0.40996 (17)	0.0337 (7)	
H4	0.8141	0.1070	0.4623	0.040*	
C5	0.5849 (4)	0.1140 (2)	0.38961 (18)	0.0393 (7)	
H5	0.5170	0.1029	0.4244	0.047*	
C6	0.5413 (3)	0.1313 (2)	0.30753 (17)	0.0311 (6)	
H6	0.4368	0.1331	0.2757	0.037*	
C7	0.6991 (3)	0.14624 (17)	0.19537 (15)	0.0220 (5)	
C8	0.5318 (3)	0.15306 (17)	0.13787 (15)	0.0229 (6)	
C9	0.4379 (3)	0.22968 (18)	0.14071 (17)	0.0292 (6)	
H9	0.4780	0.2764	0.1766	0.035*	
C10	0.2851 (3)	0.23643 (19)	0.09023 (16)	0.0316 (6)	
H10	0.2225	0.2874	0.0923	0.038*	
C11	0.2264 (3)	0.16684 (19)	0.03673 (18)	0.0325 (7)	
H11	0.1240	0.1710	0.0027	0.039*	
C12	0.3187 (3)	0.0916 (2)	0.03361 (17)	0.0329 (6)	
H12	0.2783	0.0450	−0.0023	0.039*	
C13	0.4716 (3)	0.08490 (18)	0.08371 (16)	0.0267 (6)	
H13	0.5339	0.0341	0.0808	0.032*	
C14	0.7942 (3)	0.06191 (17)	0.18337 (15)	0.0212 (5)	
C15	0.7524 (3)	−0.02053 (18)	0.21221 (17)	0.0288 (6)	
H15	0.6694	−0.0235	0.2394	0.035*	
C16	0.8343 (3)	−0.09812 (19)	0.20048 (18)	0.0356 (7)	
H16	0.8047	−0.1533	0.2190	0.043*	
C17	0.9592 (3)	−0.0945 (2)	0.16159 (16)	0.0314 (7)	
H17	1.0155	−0.1466	0.1550	0.038*	
C18	1.0000 (3)	−0.01311 (18)	0.13258 (16)	0.0291 (6)	
H18	1.0840	−0.0105	0.1061	0.035*	
C19	0.9174 (3)	0.06503 (17)	0.14234 (15)	0.0248 (6)	
H19	0.9445	0.1195	0.1214	0.030*	
N5	1.2003 (3)	0.03089 (17)	0.38553 (19)	0.0432 (7)	
O1	1.1748 (2)	0.06871 (14)	0.31497 (13)	0.0377 (5)	
O2	1.1390 (3)	0.07016 (16)	0.43733 (14)	0.0486 (6)	
O3	1.2805 (3)	−0.03780 (16)	0.4002 (2)	0.0696 (8)	
N6	1.1981 (3)	0.29851 (17)	0.42180 (14)	0.0350 (6)	
O4	1.0646 (2)	0.26855 (14)	0.43302 (12)	0.0370 (5)	
O5	1.2473 (2)	0.26008 (14)	0.36490 (13)	0.0382 (5)	
O6	1.2721 (3)	0.35837 (17)	0.46355 (14)	0.0579 (7)	

### Atomic displacement parameters (Å^2^)

**Table:**

	*U*^11^	*U*^22^	*U*^33^	*U*^12^	*U*^13^	*U*^23^
Co1	0.0188 (2)	0.0290 (3)	0.0254 (2)	−0.00224 (13)	0.00403 (16)	−0.00001 (14)
N1	0.0205 (11)	0.0212 (11)	0.0222 (11)	−0.0004 (8)	0.0047 (9)	−0.0017 (8)
N2	0.0210 (11)	0.0229 (11)	0.0272 (11)	−0.0030 (9)	0.0067 (9)	−0.0025 (9)
N3	0.0207 (11)	0.0298 (11)	0.0210 (11)	−0.0023 (9)	0.0058 (9)	−0.0030 (9)
N4	0.0220 (11)	0.0344 (12)	0.0223 (11)	−0.0030 (10)	0.0032 (9)	−0.0003 (9)
C1	0.0282 (15)	0.0282 (14)	0.0326 (15)	−0.0059 (11)	0.0102 (12)	−0.0035 (11)
C2	0.0368 (16)	0.0267 (14)	0.0337 (16)	−0.0023 (12)	0.0123 (13)	0.0057 (12)
C3	0.0329 (15)	0.0267 (14)	0.0240 (14)	0.0036 (11)	0.0072 (12)	0.0009 (11)
C4	0.0313 (15)	0.0478 (18)	0.0224 (14)	−0.0073 (13)	0.0076 (12)	−0.0004 (12)
C5	0.0329 (16)	0.059 (2)	0.0305 (16)	−0.0096 (15)	0.0170 (13)	−0.0008 (14)
C6	0.0218 (14)	0.0407 (16)	0.0330 (15)	−0.0029 (12)	0.0109 (12)	−0.0035 (13)
C7	0.0207 (13)	0.0245 (13)	0.0209 (13)	−0.0034 (10)	0.0048 (10)	−0.0021 (10)
C8	0.0206 (13)	0.0260 (13)	0.0228 (13)	0.0004 (10)	0.0065 (11)	0.0006 (10)
C9	0.0270 (14)	0.0292 (15)	0.0316 (15)	0.0006 (11)	0.0070 (12)	−0.0032 (11)
C10	0.0240 (14)	0.0357 (15)	0.0358 (16)	0.0083 (12)	0.0081 (12)	0.0009 (12)
C11	0.0221 (14)	0.0448 (18)	0.0289 (15)	−0.0013 (12)	0.0025 (12)	0.0013 (12)
C12	0.0291 (15)	0.0357 (16)	0.0319 (15)	−0.0050 (12)	0.0029 (12)	−0.0092 (12)
C13	0.0264 (14)	0.0253 (13)	0.0280 (14)	0.0016 (11)	0.0054 (11)	−0.0025 (11)
C14	0.0193 (12)	0.0233 (13)	0.0206 (12)	0.0001 (10)	0.0035 (10)	−0.0017 (10)
C15	0.0274 (14)	0.0293 (15)	0.0311 (15)	0.0018 (11)	0.0100 (12)	0.0050 (11)
C16	0.0411 (17)	0.0235 (14)	0.0426 (17)	0.0040 (12)	0.0106 (14)	0.0090 (12)
C17	0.0324 (15)	0.0278 (15)	0.0314 (15)	0.0090 (11)	0.0022 (13)	−0.0019 (11)
C18	0.0241 (13)	0.0350 (15)	0.0291 (14)	0.0019 (11)	0.0082 (12)	−0.0069 (12)
C19	0.0257 (13)	0.0238 (13)	0.0247 (13)	−0.0027 (11)	0.0055 (11)	−0.0012 (10)
N5	0.0272 (13)	0.0356 (14)	0.0631 (19)	−0.0012 (11)	0.0030 (13)	0.0115 (13)
O1	0.0304 (11)	0.0371 (11)	0.0450 (12)	0.0022 (9)	0.0073 (9)	−0.0004 (10)
O2	0.0457 (13)	0.0550 (14)	0.0452 (13)	0.0062 (11)	0.0108 (11)	0.0152 (11)
O3	0.0546 (16)	0.0409 (14)	0.112 (2)	0.0174 (12)	0.0161 (16)	0.0256 (14)
N6	0.0356 (14)	0.0373 (14)	0.0293 (13)	−0.0105 (11)	0.0020 (11)	−0.0006 (11)
O4	0.0335 (11)	0.0477 (12)	0.0304 (11)	−0.0102 (9)	0.0087 (9)	−0.0085 (9)
O5	0.0325 (11)	0.0439 (12)	0.0383 (12)	−0.0100 (9)	0.0081 (9)	−0.0036 (9)
O6	0.0704 (17)	0.0561 (14)	0.0429 (13)	−0.0357 (13)	0.0045 (12)	−0.0139 (11)

### Geometric parameters (Å, °)

**Table:**

Co1—N4	2.015 (2)	C8—C9	1.397 (4)
Co1—O1	2.054 (2)	C9—C10	1.387 (4)
Co1—N2	2.058 (2)	C9—H9	0.9300
Co1—O4	2.0841 (19)	C10—C11	1.383 (4)
Co1—O5	2.205 (2)	C10—H10	0.9300
Co1—O2	2.248 (2)	C11—C12	1.372 (4)
N1—C3	1.353 (3)	C11—H11	0.9300
N1—N2	1.372 (3)	C12—C13	1.385 (4)
N1—C7	1.488 (3)	C12—H12	0.9300
N2—C1	1.333 (3)	C13—H13	0.9300
N3—C6	1.350 (3)	C14—C19	1.387 (3)
N3—N4	1.370 (3)	C14—C15	1.390 (4)
N3—C7	1.496 (3)	C15—C16	1.383 (4)
N4—C4	1.335 (3)	C15—H15	0.9300
C1—C2	1.396 (4)	C16—C17	1.376 (4)
C1—H1	0.9300	C16—H16	0.9300
C2—C3	1.364 (4)	C17—C18	1.375 (4)
C2—H2	0.9300	C17—H17	0.9300
C3—H3	0.9300	C18—C19	1.385 (4)
C4—C5	1.382 (4)	C18—H18	0.9300
C4—H4	0.9300	C19—H19	0.9300
C5—C6	1.368 (4)	N5—O3	1.219 (3)
C5—H5	0.9300	N5—O2	1.258 (4)
C6—H6	0.9300	N5—O1	1.284 (3)
C7—C14	1.528 (3)	N6—O6	1.214 (3)
C7—C8	1.533 (3)	N6—O5	1.266 (3)
C8—C13	1.377 (4)	N6—O4	1.278 (3)
			
N4—Co1—O1	114.25 (9)	N3—C7—C8	107.4 (2)
N4—Co1—N2	89.17 (9)	C14—C7—C8	114.7 (2)
O1—Co1—N2	110.01 (8)	C13—C8—C9	119.2 (2)
N4—Co1—O4	97.15 (8)	C13—C8—C7	121.4 (2)
O1—Co1—O4	133.44 (8)	C9—C8—C7	119.4 (2)
N2—Co1—O4	103.62 (8)	C10—C9—C8	120.3 (2)
N4—Co1—O5	155.92 (9)	C10—C9—H9	119.8
O1—Co1—O5	88.71 (8)	C8—C9—H9	119.8
N2—Co1—O5	89.55 (8)	C11—C10—C9	119.5 (3)
O4—Co1—O5	59.90 (8)	C11—C10—H10	120.2
N4—Co1—O2	90.96 (9)	C9—C10—H10	120.2
O1—Co1—O2	59.74 (9)	C12—C11—C10	120.3 (3)
N2—Co1—O2	168.60 (9)	C12—C11—H11	119.9
O4—Co1—O2	87.67 (9)	C10—C11—H11	119.9
O5—Co1—O2	94.93 (8)	C11—C12—C13	120.3 (3)
C3—N1—N2	110.5 (2)	C11—C12—H12	119.8
C3—N1—C7	128.0 (2)	C13—C12—H12	119.8
N2—N1—C7	120.40 (19)	C8—C13—C12	120.3 (2)
C1—N2—N1	105.3 (2)	C8—C13—H13	119.8
C1—N2—Co1	130.24 (18)	C12—C13—H13	119.8
N1—N2—Co1	124.46 (15)	C19—C14—C15	119.3 (2)
C6—N3—N4	109.9 (2)	C19—C14—C7	121.8 (2)
C6—N3—C7	129.1 (2)	C15—C14—C7	118.9 (2)
N4—N3—C7	119.0 (2)	C16—C15—C14	120.0 (2)
C4—N4—N3	105.7 (2)	C16—C15—H15	120.0
C4—N4—Co1	128.17 (18)	C14—C15—H15	120.0
N3—N4—Co1	124.22 (16)	C17—C16—C15	120.6 (3)
N2—C1—C2	110.9 (2)	C17—C16—H16	119.7
N2—C1—H1	124.5	C15—C16—H16	119.7
C2—C1—H1	124.5	C18—C17—C16	119.4 (2)
C3—C2—C1	105.5 (2)	C18—C17—H17	120.3
C3—C2—H2	127.2	C16—C17—H17	120.3
C1—C2—H2	127.2	C17—C18—C19	120.8 (2)
N1—C3—C2	107.7 (2)	C17—C18—H18	119.6
N1—C3—H3	126.1	C19—C18—H18	119.6
C2—C3—H3	126.1	C18—C19—C14	119.9 (2)
N4—C4—C5	110.8 (2)	C18—C19—H19	120.1
N4—C4—H4	124.6	C14—C19—H19	120.1
C5—C4—H4	124.6	O3—N5—O2	123.3 (3)
C6—C5—C4	105.6 (3)	O3—N5—O1	121.2 (3)
C6—C5—H5	127.2	O2—N5—O1	115.4 (2)
C4—C5—H5	127.2	N5—O1—Co1	96.51 (17)
N3—C6—C5	107.9 (2)	N5—O2—Co1	88.28 (16)
N3—C6—H6	126.0	O6—N6—O5	123.1 (3)
C5—C6—H6	126.0	O6—N6—O4	122.1 (3)
N1—C7—N3	108.57 (19)	O5—N6—O4	114.8 (2)
N1—C7—C14	109.52 (19)	N6—O4—Co1	95.26 (15)
N3—C7—C14	107.9 (2)	N6—O5—Co1	90.03 (15)
N1—C7—C8	108.6 (2)		
			
C3—N1—N2—C1	2.5 (3)	C14—C7—C8—C13	3.7 (4)
C7—N1—N2—C1	171.1 (2)	N1—C7—C8—C9	−53.9 (3)
C3—N1—N2—Co1	−177.56 (16)	N3—C7—C8—C9	63.3 (3)
C7—N1—N2—Co1	−8.9 (3)	C14—C7—C8—C9	−176.7 (2)
N4—Co1—N2—C1	157.3 (2)	C13—C8—C9—C10	0.8 (4)
O1—Co1—N2—C1	−87.1 (2)	C7—C8—C9—C10	−178.8 (2)
O4—Co1—N2—C1	60.2 (2)	C8—C9—C10—C11	−0.3 (4)
O5—Co1—N2—C1	1.4 (2)	C9—C10—C11—C12	0.0 (4)
O2—Co1—N2—C1	−111.9 (4)	C10—C11—C12—C13	−0.3 (4)
N4—Co1—N2—N1	−22.63 (19)	C9—C8—C13—C12	−1.0 (4)
O1—Co1—N2—N1	92.95 (19)	C7—C8—C13—C12	178.6 (2)
O4—Co1—N2—N1	−119.78 (18)	C11—C12—C13—C8	0.8 (4)
O5—Co1—N2—N1	−178.58 (19)	N1—C7—C14—C19	−19.7 (3)
O2—Co1—N2—N1	68.1 (5)	N3—C7—C14—C19	−137.7 (2)
C6—N3—N4—C4	−1.9 (3)	C8—C7—C14—C19	102.6 (3)
C7—N3—N4—C4	−167.1 (2)	N1—C7—C14—C15	162.4 (2)
C6—N3—N4—Co1	−167.12 (19)	N3—C7—C14—C15	44.4 (3)
C7—N3—N4—Co1	27.7 (3)	C8—C7—C14—C15	−75.3 (3)
O1—Co1—N4—C4	99.7 (3)	C19—C14—C15—C16	0.5 (4)
N2—Co1—N4—C4	−148.7 (3)	C7—C14—C15—C16	178.6 (2)
O4—Co1—N4—C4	−45.1 (3)	C14—C15—C16—C17	1.1 (4)
O5—Co1—N4—C4	−61.7 (3)	C15—C16—C17—C18	−1.5 (4)
O2—Co1—N4—C4	42.7 (3)	C16—C17—C18—C19	0.3 (4)
O1—Co1—N4—N3	−98.5 (2)	C17—C18—C19—C14	1.4 (4)
N2—Co1—N4—N3	13.1 (2)	C15—C14—C19—C18	−1.8 (4)
O4—Co1—N4—N3	116.75 (19)	C7—C14—C19—C18	−179.7 (2)
O5—Co1—N4—N3	100.1 (3)	O3—N5—O1—Co1	178.8 (2)
O2—Co1—N4—N3	−155.5 (2)	O2—N5—O1—Co1	−2.1 (3)
N1—N2—C1—C2	−1.5 (3)	N4—Co1—O1—N5	−74.82 (17)
Co1—N2—C1—C2	178.50 (18)	N2—Co1—O1—N5	−173.28 (15)
N2—C1—C2—C3	0.1 (3)	O4—Co1—O1—N5	53.1 (2)
N2—N1—C3—C2	−2.5 (3)	O5—Co1—O1—N5	97.67 (16)
C7—N1—C3—C2	−170.1 (2)	O2—Co1—O1—N5	1.20 (15)
C1—C2—C3—N1	1.4 (3)	O3—N5—O2—Co1	−179.0 (3)
N3—N4—C4—C5	1.1 (3)	O1—N5—O2—Co1	1.9 (2)
Co1—N4—C4—C5	165.6 (2)	N4—Co1—O2—N5	116.54 (17)
N4—C4—C5—C6	0.0 (4)	O1—Co1—O2—N5	−1.22 (15)
N4—N3—C6—C5	1.9 (3)	N2—Co1—O2—N5	26.0 (5)
C7—N3—C6—C5	165.2 (3)	O4—Co1—O2—N5	−146.35 (17)
C4—C5—C6—N3	−1.1 (3)	O5—Co1—O2—N5	−86.83 (17)
C3—N1—C7—N3	−138.1 (2)	O6—N6—O4—Co1	−178.9 (3)
N2—N1—C7—N3	55.4 (3)	O5—N6—O4—Co1	0.1 (2)
C3—N1—C7—C14	104.3 (3)	N4—Co1—O4—N6	−172.34 (16)
N2—N1—C7—C14	−62.2 (3)	O1—Co1—O4—N6	54.13 (19)
C3—N1—C7—C8	−21.6 (3)	N2—Co1—O4—N6	−81.46 (17)
N2—N1—C7—C8	171.9 (2)	O5—Co1—O4—N6	−0.08 (14)
C6—N3—C7—N1	131.7 (3)	O2—Co1—O4—N6	96.98 (16)
N4—N3—C7—N1	−66.4 (3)	O6—N6—O5—Co1	178.9 (3)
C6—N3—C7—C14	−109.7 (3)	O4—N6—O5—Co1	−0.1 (2)
N4—N3—C7—C14	52.3 (3)	N4—Co1—O5—N6	19.2 (3)
C6—N3—C7—C8	14.4 (4)	O1—Co1—O5—N6	−143.82 (16)
N4—N3—C7—C8	176.4 (2)	N2—Co1—O5—N6	106.16 (16)
N1—C7—C8—C13	126.5 (2)	O4—Co1—O5—N6	0.08 (14)
N3—C7—C8—C13	−116.3 (3)	O2—Co1—O5—N6	−84.34 (16)

### Hydrogen-bond geometry (Å, °)

**Table:**

*D*—H···*A*	*D*—H	H···*A*	*D*···*A*	*D*—H···*A*
C17—H17···O5^i^	0.93	2.54	3.413 (3)	157
C10—H10···O3^ii^	0.93	2.59	3.399 (4)	146
C3—H3···O4^iii^	0.93	2.50	3.313 (3)	146
C19—H19···N1	0.93	2.46	2.799 (3)	102

Symmetry codes: (i) −*x*+5/2, *y*−1/2, −*z*+1/2; (ii) −*x*+3/2, *y*+1/2, −*z*+1/2; (iii) *x*−1/2, −*y*+1/2, *z*−1/2.

## References

[bb1] Baho, N. & Zargarian, D. (2007*a*). *Inorg. Chem.* **46**, 299–308. 10.1021/ic061311z 17198440

[bb2] Baho, N. & Zargarian, D. (2007*b*). *Inorg. Chem.* **46**, 7621–7632. 10.1021/ic070093m 17665903

[bb3] Bruker (2008). *XPREP* Bruker AXS Inc., Madison, Wisconsin, USA.

[bb30] Bruker (2009). *APEX2* and *SAINT* Bruker AXS Inc., Madison, Wisconsin, USA.

[bb4] Reger, D. L., Gardinier, J. R. & Smith, M. D. (2004). *Inorg. Chem.* **43**, 3825–3832. 10.1021/ic0497174 15206862

[bb5] Shaw, J. L., Cardon, T., Lorigan, G. & Ziegler, C. J. (2004). *Eur. J. Inorg. Chem.* **5**, 1073–1080.

[bb6] Shaw, J. L., Gwaltney, K. P. & Keer, N. (2009). *Inorg. Chim. Acta*, **362**, 2396–2401.

[bb7] Shaw, J. L., Yee, G. T., Wang, G. W., Benson, D. E., Gokdemir, C. & Ziegler, C. J. (2005). *Inorg. Chem.* **44**, 5060–5067. 10.1021/ic048229t 15998034

[bb8] Sheldrick, G. M. (2008*a*). *SADABS* University of Göttingen, Germany.

[bb9] Sheldrick, G. M. (2008*b*). *Acta Cryst.* A**64**, 112–122. 10.1107/S0108767307043930 18156677

[bb10] Shiu, K., Yeh, L., Peng, S. & Cheng, M. (1993). *J. Organomet. Chem.* **460**, 203–211.

[bb11] Tsuji, S., Swenson, D. C. & Jordan, R. F. (1999). *Organometallics* , **18**, 4758–4764.

[bb12] Westrip, S. P. (2010). *publCIF* In preparation.

